# An Unexpected Factor to Wellens Syndrome

**DOI:** 10.7759/cureus.14489

**Published:** 2021-04-14

**Authors:** Billal Mohmand, Abeeha Naqvi, Anojan Pathmanathan, Mark Charlamb

**Affiliations:** 1 Medicine, Upstate University Hospital, Syracuse, USA; 2 Internal Medicine, Upstate University Hospital, Syracuse, USA; 3 Cardiology, Upstate University Hospital, Syracuse, USA

**Keywords:** wellens' syndrome, heterozygous factor v leiden, left anterior descending artery, coronary artery occlusion, genetics of coronary artery disease, factor v, coronary artery angiography, risk factors cardiovascular diseases

## Abstract

Factor V Leiden deficiency, the most common inherited thrombophilia, is a risk factor for venous thromboembolism in both the heterozygous and homozygous forms. An autosomal dominant genetic condition, the pattern of incomplete penetrance leads to variable manifestations of the disease. The association with arterial thromboembolism remains controversial, with studies indicating modest increases in risk of coronary artery disease, stroke. We present the case of a 53-year-old male with Wellens syndrome, with a history of heterozygous factor V Leiden deficiency and no other risk factors. Coronary angiography found a complete total occlusion of the proximal left anterior descending artery, with established collaterals, ultimately requiring coronary artery bypass graft. Laboratory testing effectively eliminated the presence of any alternative known risk factors for the advanced coronary artery disease. The literature evaluating a possible link between factor V Leiden deficiency and atherosclerosis remain conflicting. Our case highlights a concerning association and need for further studies.

## Introduction

Wellens syndrome or left anterior descending (LAD) artery T wave inversion syndrome is an electrocardiographic abnormality that was initially described in 1982 [1]. It is found to be highly specific for identifying critical proximal LAD artery stenosis.

Its incidence is estimated around 10%-15% of acute coronary syndrome presentations and is often under-recognized. The diagnostic criteria include two characteristic electrocardiogram (ECG) patterns [2]. Type A presents as symmetric biphasic T waves in precordial leads V2 and V3 and is a specific but less common and difficult-to-recognize finding. Type B presents as deep, symmetric inverted T waves in anterior leads, which is less specific. T wave changes may be transient, persist for months, or completely resolve with revascularization. These combined findings have an 86% positive predictive value [3]. These critical features represent a pre-infarction state of coronary artery disease (CAD) with significant risk for myocardial infarction. As the condition is a manifestation of CAD, the typical risk factors include hypertension, diabetes, hyperlipidemia, obesity, smoking, and familial history. However, further risk factors for CAD have been suggested with variable evidence. Hypercoagulable states due to genetic mutations have been suggested by multiple studies. 

Factor V Leiden (FVL), also known as factor VR506Q and factor V Arg506 Gln, results from a single-point mutation in the factor V gene (guanine to adenine, at nucleotide 1691), which leads to a single amino acid change (replacement of arginine with glutamine at amino acid 506) [4]. The subsequent protein is resistant to cleave by activated Protein C, leading to a hypercoagulable state. The direct effect on CAD has been controversial. There are documented cases suspecting an association between the mutation and myocardial infarctions. Additionally, investigations have indicated association with coronary atherosclerosis. Our case describes a middle-aged male, a sole risk factor of heterozygous FVL deficiency, who was found to have Wellens Type B pattern on ECG in the emergency department.

## Case presentation

A 53-year-old Caucasian male with a past medical history significant only for heterozygous FVL deficiency presented with a complaint of typical angina of two weeks duration. The patient reported an athletic lifestyle, with recent exertional chest pain while kayaking and cycling. The patient reported that his most recent episode of pain awoke him from his sleep, prompting him to seek evaluation in the emergency department.

**Figure 1:**
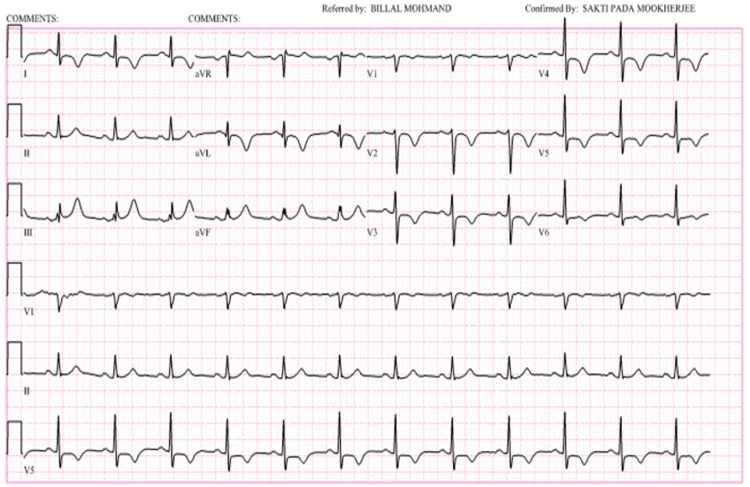
Emergency department electrocardiogram indicating sinus rhythm with deep symmetrical T wave inversions across anterior precordial leads suggestive of Wellens syndrome with Type B findings

Laboratory testing revealed an elevated troponin I of 0.43 ng/mL (normal: <0.08 ng/mL), troponin T of 0.13 ng/mL (normal: <0.01 ng/mL), and pro B-type natriuretic peptide of 898 pg/mL (normal: <125 pg/mL). ECG showed normal sinus rhythm with deep symmetrical T wave inversions across anterior precordial leads suggestive of Wellens syndrome with Type B findings (Figure 1).

Significant risk factors were evaluated and not found to be present. The patient was found to have a lipid profile of cholesterol 145 mg/dL, high-density lipoprotein (HDL) 32 mg/dL, low-density lipoprotein 95 mg/dL, non-HDL 113 mg/dL, triglycerides 88 mg/dL, very low-density lipoprotein 18 mg/dL, and hemoglobin A1c of 5.6%. The patient denied any history of hypertension and presented with a blood pressure of 122/79 mmHg on admission. He was recorded to have a body mass index of 26.3 kg/m^2^ and denied any smoking or alcohol history. No familial history of premature CAD was present. The patient was considered a moderate risk based on the HEART Score for six-week risk of major adverse cardiac events.

**Figure 2:**
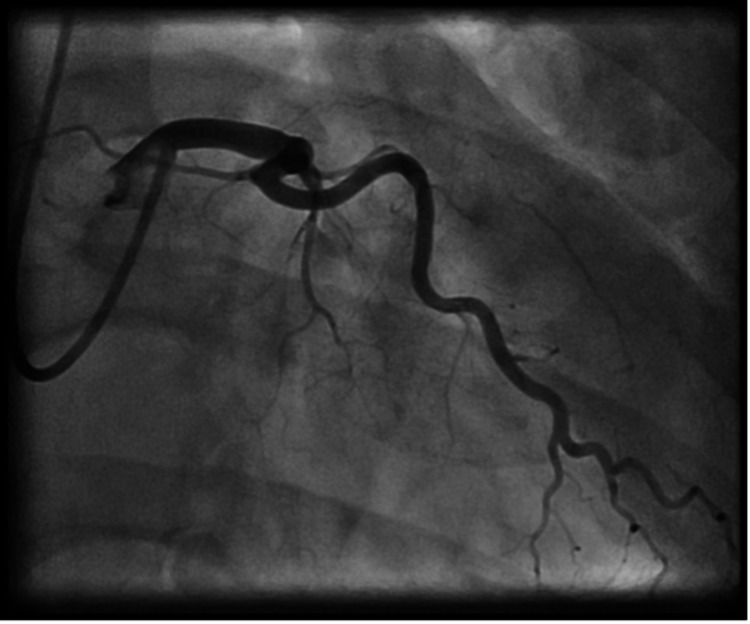
Complete ostial occlusion of the left anterior descending artery. Left circumflex artery with no angiographically significant disease

**Figure 3:**
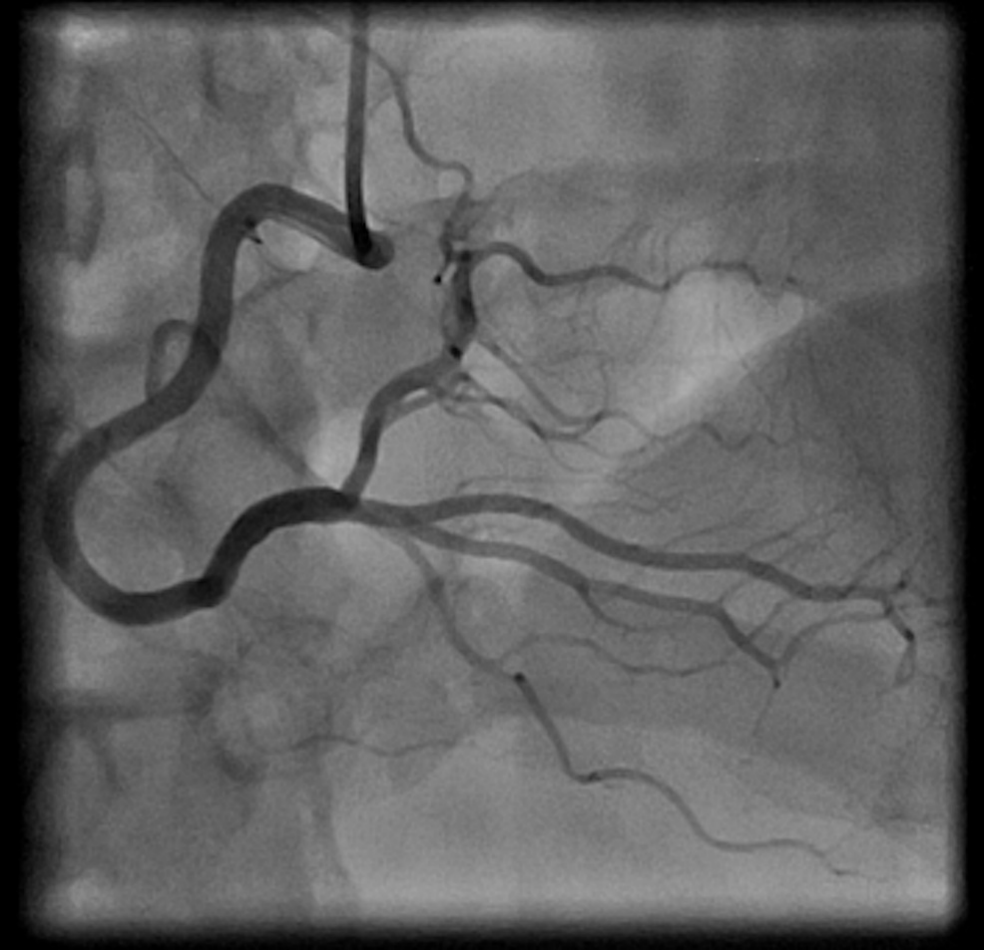
Right coronary artery with no angiographically significant disease

**Figure 4:**
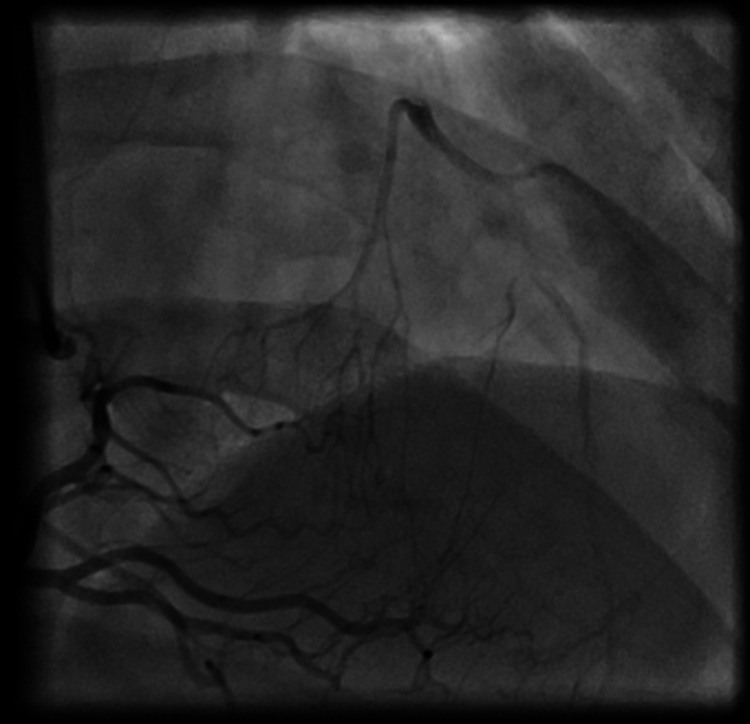
Well-established collateral arteries from right coronary artery supplying the midsegment of the left anterior descending artery

The patient underwent emergent cardiac catheterization, which revealed one-vessel CAD with a complete ostial occlusion of the LAD (Figure 2). The right coronary artery (RCA) and left circumflex artery did not have any angiographically significant disease. Well-established collaterals were present from the dominant RCA, which supplied the mid-segment of the LAD (Figures 3, 4). Due to the proximal location and nature of the occlusion, no intervention was attempted, and the patient was immediately referred to cardiac surgery for coronary artery bypass graft (CABG).

The patient underwent successful CABG with the left internal mammary artery to the LAD. He was ultimately discharged home on guideline-directed medical therapy, dual anti-platelet therapy with aspirin and clopidogrel, and without any complications from the procedure.

## Discussion

Our case presents a middle-aged Caucasian male with no significant cardiac risk factors or CAD. Interestingly, his history was remarkable for heterozygous FVL deficiency. Despite the patient’s athletic lifestyle and lack of risk factors, the patient presented with a critical proximal occlusion of the LAD consistent with the ECG findings of Type B Wellens syndrome. 

The association with arterial thromboembolism remains controversial, with studies indicating modest increases in risk of CAD, stroke. In our case, a detailed history and laboratory testing effectively eliminated the presence of any typical risk factors for the advanced CAD. The literature evaluating a possible link between FVL deficiency and atherosclerosis remain controversial. 

A genotypic evaluation of polymorphisms suspected FVL of contributing to the development of CAD. Patients with preexisting CAD were evaluated and diabetic and hypertensive patients were excluded. Of 36 men and 22 women enrolled with an age ranging between 41 and 85 (mean age: 62.75 ± 9.18 years), heterozygous FVL genotype was found in eight (13.8%) patients (six males and two females) [5]. FVL was suspected of contributing to the development of CAD.

FVL is the most common inherited thrombophilia with a prevalence of 1%-7% in the Caucasian population [6]. FVL deficiency may contribute to initial development of CAD; however, screening and testing remain a controversial topic. Our case highlights an association between FVL deficiency and significant CAD despite lack of other significant risk factors.

## Conclusions

The possibility of an increased risk of CAD in the population with FVL could have significant impact on prevention methods, targeted testing, and treatment. Critical analysis of the role of FVL deficiency toward the pathogenesis of CAD remains imperative. The association established in our case and in multiple others emphasizes the need for further investigation and consideration for management of these patients.
